# Exercise Testing in Aortic Stenosis: Safety, Tolerability, Clinical Benefits and Prognostic Value

**DOI:** 10.3390/jcm11174983

**Published:** 2022-08-25

**Authors:** Sahrai Saeed, John B. Chambers

**Affiliations:** 1 Department of Heart Disease, Haukeland University Hospital, 5021 Bergen, Norway; 2 Cardiothoracic Centre, Guy’s and Saint Thomas’ Hospital, London SE1 9RS, UK

**Keywords:** aortic stenosis, exercise testing, treadmill exercise, valvular heart disease

## Abstract

Background: Routine exercise testing in asymptomatic patients with valvular heart disease (VHD) better classifies the hemodynamic severity of valve stenosis or regurgitation, and describes the symptomatic status and functional capacity of the patient. This is crucial for planned surveillance and optimal timing of surgery, particularly for aortic stenosis (AS), because once symptoms occur, there is a sharp increase in the risk of sudden death unless valve intervention is performed. Purpose: To conduct a focused clinical review on the benefits of exercise testing in patients with AS. Methods: The electronic database PubMed was systematically searched for relevant retrospective and prospective cohort studies reporting on the safety, feasibility and tolerability of exercise testing in VHD, with a special focus on AS. Results and conclusions: In patients with significant AS, exercise testing is safe, feasible and reveals symptoms in a significant proportion of patients. In addition, serial testing has incremental prognostic value over a baseline test alone. Exercise testing in patients with AS is underused and should be performed routinely to refine the hemodynamic severity of AS.

## 1. Introduction

Current European Society of Cardiology and American College of Cardiology/American Heart Association guidelines on the management of valvular heart disease (VHD) recommend exercise testing in patients who are apparently asymptomatic or have equivocal symptoms [1,2]. Exercise testing in asymptomatic patients with VHD better classifies the hemodynamic severity of valve stenosis or regurgitation, and describes the symptomatic status and functional capacity of the patient to optimize the timing of surgery. This is of particular importance for aortic stenosis (AS), because once symptoms occur there is a sharp increase in the risk of sudden death unless valve intervention is performed [3,4,5,6]. In addition, physiological measures during exercise testing may help in risk assessment [5,7]. However, in the EuroHeart survey, exercise testing was performed in <10% of those in whom it was indicated [8]. A study from Western Norway showed that only 15% of asymptomatic patients with severe AS had undergone an exercise test and one-third of the patients who were apparently asymptomatic developed symptoms during exercise testing [9]. 

The underuse of exercise testing in AS may be due to concerns over safety and tolerability, or for logistic reasons, or because comorbidities might make interpretation difficult [8,9,10,11]. It is likely that the underuse of exercise testing contributes to patients presenting with advanced symptoms. Approximately one half of patients with all types of valve disease in the EuroHeart Survey had grade III or IV symptoms at surgery [8]. A recent survey of clinical characteristics of patients with AS showed that 40% had grade III or IV symptoms at presentation [12]. Hence, the aim of this clinical review is to highlight the benefits of exercise testing in patients with AS. 

## 2. Methods

The electronic database PubMed was systematically searched for relevant original studies in English reporting on the safety, feasibility, tolerability of exercise testing and assessing the symptomatic status of patients with VHD, with a special focus on AS. The search was conducted between November 2021 and December 2021. The keywords used were “Aortic Stenosis”, “Treadmill exercise” and “Exercise testing”.

## 3. How Is Exercise Testing Performed?

Recommendations on exercise testing in VHD were last updated in the US in 2002 [7]. Since then, there have been some focused expert updates on exercise testing in aortic valve disease [6,13,14]. Exercise may be performed using a bicycle or treadmill. In severe aortic or mitral regurgitation, a standard Bruce protocol is used, but in AS a modified Bruce protocol with two warm-up stages with medical or nursing supervision is usually recommended [5,15,16,17]. In most Scandinavian countries, the exercise test in AS patients is often referred to as “low threshold exercise test” or “a submaximal stress test” with a lower workload intensity than the standard used for coronary artery disease (CAD). A bicycle is the preferred exercise modality. Table 1 illustrates standard hemodynamic parameters and the 12-lead ECG measures to be recorded at baseline, end of each stage, and at peak exercise.

The criteria for an abnormal exercise test are presented in Table 2 [5,10,14,16,18].

In the past, AS was a contraindication to exercise testing; however recent studies, comprising of a total of 1850 tests [16,19,20,21] in patients who were asymptomatic, showed good tolerability with no adverse clinical events. Even in symptomatic patients, exercise testing under careful hemodynamic monitoring is safe [22,23,24], although these have been performed for research rather than clinical reasons. Exercise testing may not be appropriate in patients in whom a valve intervention is indicated only for significant symptoms, for example, for the elderly or those at high surgical risk, or where other pathology limits exercise (e.g., chronic obstructive pulmonary disease or anemia). 

## 4. Symptoms

The mortality in asymptomatic AS is <1% per year, but patients typically slow down to avoid symptoms [9]. They may be at increased risk in this period, but certainly the mortality increases precipitously with the development of spontaneous symptoms, which is a critical point in the natural history of AS. The mortality is 3–4% within three months of the onset of symptoms [5,25], and may be as high as 14% on a six-month surgical waiting list. Exercise testing reveals symptoms in patients just before this precipitous rise in mortality and allows surgery to be expedited [5]. 

Symptoms are revealed in approximately 40% of patients with asymptomatic severe AS and 24% with moderate AS [16]. The reason for positive results in moderate AS include: the arbitrary cut-point between high-end moderate and severe; reduced valve compliance; associated coronary artery disease; increased vascular stiffness resulting in high total left ventricular (LV) outflow impedance; LV dysfunction for other reasons including amyloidosis or prior systemic hypertension. This may also explain why the hemodynamic severity of significant valve stenosis does not differ between patients with or without symptoms [9]. Patients with revealed symptoms have a high risk of events [16,18,19,26]. In the exercise testing in AS (EXTAS) study, 2-year event-free survival with revealed symptoms was nearly 46% versus 70% in those without revealed symptoms [16]. A detailed description of the clinical outcome, prevalence of abnormal exercise tests, and major findings in the relevant AS studies are presented in Table 3 [5,16,20,21,22,23,24,26,27,28,29,30,31,32,33,34,35,36,37,38]. 

## 5. Physiological Measurements

Physiological measurements may add important information to revealed symptoms because of the concerns that revealed symptoms alone may be subjective and reduce its predictive value in elderly subjects [31]. A gradual and appropriate increase in heart rate and blood pressure (BP) within the physiological range reflect good functional capacity, as reflected by METs or exercise duration. Although an abnormal BP response is usually defined by a blunted rise, recent data show that an abnormal (exaggerated) increase may also be prognostically useful [39]. The heart rate (HR) rise has also been explored [40]. ST segment changes have not been shown to be useful. Some studies recommend ≥2 mm ST-segment depression in comparison to baseline levels [31,41]. However, ST segment depression has repeatedly been shown to be non-specific in AS patients who have a relatively high prevalence of hypertension and LV hypertrophy (LVH), and should not be used as a criterion of positivity alone. In a recent EXTAS sub-study, the prevalence of ST segment depression (>5 mm) was 13% in the total study population, which was comparable in patients with and without revealed symptoms (17.6% versus 11.3%, *p* = 0.132) [42]. ST segment depression on ETT was strongly associated with aortic valve area. According to univariate Cox regression analyses, ST segment depression was not associated with cardiac related hospitalizations, AVR, or all-cause mortality. 

## 6. Blunted BP Rise

A blunted BP rise may be defined in different ways: (1) a sustained fall in systolic BP ≥ 20 mmHg below the previous stage, (2) a fall 20 mmHg below the baseline level, or (3) a failure to rise more than 25% from the baseline level [10,14,39]. The 2017 ESC guidance does not provide a clear definition of blunted BP response, but recommend (class IIa) aortic valve intervention in asymptomatic patients with severe AS and an abnormal exercise test showing a decrease in BP below baseline level [2].

## 7. Exaggerated BP Response

In the general population, an exaggerated BP response is associated with a greater risk of incident hypertension, masked hypertension [43,44,45,46], and cardiovascular morbidity and mortality [47,48]. There is little evidence in AS and no consensus on the definition. In one study, a peak systolic BP level >190 mmHg was used [39]. The peak systolic BP during the exercise test was dependent on the patient’s age, the level of the resting BP prior to exercise test, and exercise intensity, and also increased LV mass and systemic arterial stiffness. However, it could not predict adverse outcomes. Unlike in the general population, an exaggerated BP response is not yet a useful index in AS.

## 8. Abnormal Heart Rate Response to Exercise

An early rapid rise in heart rate (RR-HR) is associated with revealed symptoms later in the test and increases the risk of death in moderate AS in the following two years [40]. RR-HR is defined as achieving at least 85% of target HR or a ≥50% increase from baseline within the first 6 min. The likely explanation of RR-HR is as a compensation for the fall in stroke volume to maintain cardiac output in early exercise that occurs in patients with spontaneous or revealed symptoms. By comparison, in asymptomatic patients, the stroke volume rises in early exercise. Thus, in view of this study, a normal HR response to exercise tests may be reassuring when the presenting symptoms are doubtful. 

Chronotropic incompetence, defined as a blunted increase in HR during exercise, relates to worse symptomatic status and diminished exercise capacity in a variety of clinical entities [49]. However, in patients with AS patients who are often elderly, with increased arterial stiffness [50,51], and relatively frequent use of β blockers [52,53], a blunted HR increase may have limited clinical and prognostic importance. The data on a blunted HR increase in AS patients is scarce and should be explored in future prospective studies. 

## 9. Serial Testing

**Figure 1:**
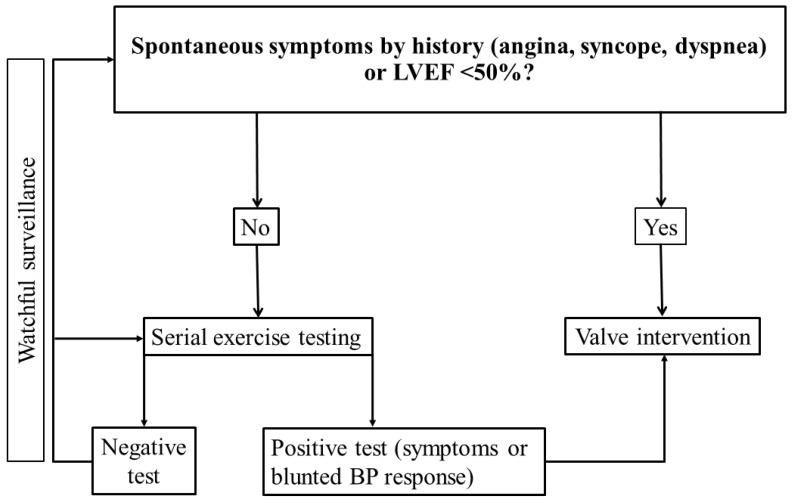
Management of aortic stenosis and the role of exercise testing in watchful waiting. BP, blood pressure; LVEF, left ventricular ejection fraction.

A negative exercise test has excellent prognostic value, and a watchful surveillance is only safe if the patients remain asymptomatic on exercise testing (Figure 1). Few data exist on the role of serial exercise testing in AS. It is known that serial exercise testing adds incremental prognostic value to baseline testing. In the EXTAS cohort study, over a follow-up period of 35 months, 59% of patients experienced revealed symptoms at some point during serial ETTs [16]. When revealed symptoms were added to aortic valve area and peak aortic jet velocity, the area under the curve increased from 0.74 to 0.79 (*p* = 0.01) with an additional 26% improvement of risk classification for the composite endpoint of AVR and all-cause mortality, but not for all-cause mortality alone. In addition, serial exercise testing may identify patients with declining functional capacity over time. 

## 10. Exercise Stress Testing with Imaging 

Exercise echocardiography may help in identifying the cardiac source of dyspnoea. However, the evidence for its prognostic value is confined mainly to AS and mitral regurgitation [14,17,54]. A number of studies have shown that exercise echocardiography in AS is safe and feasible, and provides important clinical and prognostic information [14,17,20,22,23,28,29,31,33,35,36,55]. Maréchaux et al. demonstrated the usefulness of exercise stress echocardiography for risk stratification in asymptomatic patients with severe AS [33]. Patients with a fall in LV ejection fraction (EF) on exercise were more likely to develop symptoms than those in whom the LVEF rose on exercise [29]. Hence, exercise echocardiography provides a simultaneous assessment of exercise-induced symptoms and hemodynamic changes (increase in mean pressure gradient, improvement or deterioration of LVEF and contractile reserve, increase in filling pressure and systolic pulmonary artery pressure) [1,2,14,17]. 

Exercise echocardiography can be combined with a treadmill, but the images are acquired immediately after the stress and not at peak stress. Thus, HR and the pattern of LV response may change quickly after stopping exercise. By contrast, simultaneous echocardiography with supine/semi-supine bicycle exercise echocardiography offers a direct assessment of LV response to exercise and rules out exercise-induced regional wall motion abnormalities, diastolic dysfunction, and pulmonary hypertension, which are associated with a more advanced stage of AS and worse prognosis [36]. Similarly, it can also exclude a biphasic LV response to exercise, e.g., initial improvement of EF and myocardial contractility at low intensity workload, but decline later in high intensity workload [56]. 

Finally, pharmacologic stress echocardiography with dobutamine is indicated in patients with low gradient and low EF severe AS to determine the grade of AS and the LV contractile reserve. Severe AS is defined by a mean gradient of 40 mmHg occurring at any time during the test [1,2,57]. Contractile reserve is defined by an increase in EF, stroke volume, or subaortic VTI by 20%. Patients without contractile reserve have a substantially higher operative mortality than those with contractile reserve [58].

## 11. Conclusions

In patients with significant aortic stenosis, exercise testing is safe, feasible, and reveals symptoms in a considerable amount of patients. Serial testing has incremental prognostic value over baseline testing. Exercise testing in patients with aortic stenosis is underused and should be more often performed at valve clinics.

## Figures and Tables

**Table 1:** Standard hemodynamic parameters and 12-lead ECG measures to be recorded at baseline, end of each stage, and at peak exercise.

**Hemodynamic parameters**
Pre-exercise heart rate
Pre-exercise blood pressure
Peak heart rate
Peak blood pressure
Post-exercise blood pressure
Exercise duration
Exercise test stage at stopping
Reason for stopping including symptoms
Metabolic equivalents
**ECG measures to be monitored**
ST segment depression
Premature ventricular contraction
Arrhythmias (supraventricular tachycardia, atrial fibrillation)
Target heart rate achieved?

**Table 2:** Criteria of an abnormal exercise test.

**Limiting symptoms:**
Significant breathlessness
Angina (chest constriction/tightness)
Dizziness
**Ischemic ST segment changes and BP response:**
≥3–5 mm ST segment depression
Blunted BP response (variously defined as failure of systolic BP to rise >25%; or a sustained fall in systolic BP > 20 mmHg from the previous stage or below the baseline level)
**Sustained tachyarrhythmias:**
Progressive ventricular arrhythmias >3 beats
New onset atrial fibrillation
**Reduced functional capacity:**
Maximal exhaustion at low workload (or progressive decline in serial testing)
**Abnormal heart rate response:**
An early rapid rise in heart rate to at least 85% of target heart rate or a ≥50% increase from baseline within the first 6 min

BP, blood pressure.

**Table 3:** Exercise stress test studies in patients with moderate or severe aortic stenosis patients.

First Author, Year [Ref]	Study Design and Follow-Up	No. of Pts.	Age and Gender	Exercise Modeand Protocol	Exercise Echo	Clinical Events and Major Findings
Amato et al., 2001 [27]	Prospective 15 ± 12 months	66	50 ± 15 years 67% men	Treadmill (Ellestad)	−	Exercise test was safe. Patients with positive stress test (67%) had a 7.6-fold increased risk of developing symptoms or sudden death at follow-up.
Alborino et al., 2002 [26]	Prospective 36 months	30	62 ± 14 years 67% men	Upright bicycle 25 W + 10–50 W 2nd min	−	Exercise test was safe. An abnormal test was found in 60% of patients.
Das et al., 2005 [5]	Prospective 12 months	125	56–74 years 68% men	Treadmill (modified Bruce)	−	Exercise test was safe and revealed symptoms in 37% of patients.
Lancellotti et al., 2005 [28]	Prospective 15 ± 7 months	69	66 ± 12 years 70% men	Semi-supine bicycle (25 + 25 W 2nd min)	+	Exercise test was safe. Abnormal exercise test was observed in 26% of patients.
Maréchaux et al., 2007 [29]	Prospective 11 (2–40) months	50	65 ± 13 years 54% men	Semi-supine bicycle ergometer (25 + 25 W)	+	Abnormal LV response (11% fall in mean EF to exercise) was found in 40% of patients. These were more likely to develop symptoms compared to those who showed a rise in EF on exercise.
Peidro et al., 2007 [30]	Prospective 11 (5–19) months	102	64 ± 14 years 61% men	Treadmill (Naughton)	−	Exercise test was safe. Exercise test was abnormal in 65.7% of patients.
Lancellotti et al., 2008 [31]	Prospective Cross-sectional	128		Semi-supine bicycle on a tilting table (25 W + 25 W each 2nd min)	+	Exercise test was abnormal in 47% of patients, and mediated by larger increase in mean gradient and decrease/smaller increase in LV ejection fraction.
Lafitte et al., 2009 [32]	Prospective 12 months	65 pts 60 controls	70 ± 12 years 82% men, 66 ± 15 years 75% men	Treadmill Bruce (modified by 2 warm-up stages)	−	Exercise test was abnormal in 65% of patients. Impaired global longitudinal strain assessed by 2D was associated with abnormal exercise test and higher risk of cardiac events during follow-up.
Laskey et al., 2009 [24]	Cross-sectional	18 pts 11 controls	60 ± 8 years 72% men, 53 ± 7 years 64% men	Supine bicycle (25 W + 25 W)	−	Compared with control subjects, patients with AS showed reduced arterial compliance and increased systemic vascular resistance at rest, but a further arterial stiffening and blunted increase in flow rate during exercise.
Maréchaux et al., 2010 [33]	Retrospective 20 ± 14 months	186	64 ± 15 years 64% men	Semi-supine bicycle 20–25 W + 20–25 W each 3rd min)	+	Exercise test was abnormal in 27% of patients. Exercise echocardiography provided additional prognostic information.
Rajani et al., 2010 [34]	Prospective Cross-sectional	38	63 (29–83) years, 84% men	Treadmill Bruce (modified by 2 warm-up stages)	−	Symptoms were revealed in 26% of patients and associated with lower peak myocardial VO2, stroke index, and a trend towards a blunted fall in systemic vascular resistance. BNP was the strongest resting predictor of revealed symptoms.
Dalsgaard et al., 2010 [21]	Prospective Cross-sectional	29	69 ± 8 years 66% men	CPET with multistage supine bicycle (25 W + 25 W each 2nd min)	−	Exercise test was safe. Symptoms were revealed in 69% of patients. The marker of diastolic dysfunction were closely related to the severity of AS.
Donal et al., 2011 [35]	Prospective Cross-sectional	207 pts 43 control subjects	67 ± 11 years 66% men 68 ± 11 years 71% men	Graded semi-supine bicycle on tilting table (30 W + 20 W 2nd min)	+	Exercise test was abnormal in 34% of patients. Reduced longitudinal myocardial function and missing contractile reserve during exercise in spite of normal EF at rest.
Lancellotti et al., 2012 [36]	Prospective Cross-sectional	105	71 ± 9 years 59% men	Semi-supine bicycle on a tilting table (25 W + 25 W each 2nd min)	+	Exercise pulmonary hypertension was found in 55% of patients and was associated with a 2-fold increased risk of cardiac events. Male gender, resting SPAP, and measures of diastolic dysfunction during exercise were the main determinants of exercise pulmonary hypertension.
Dulgheru et al., 2013 [37]	Cross-sectional	62	65 ± 13 years 68% men	Treadmill CPET (modified Bruce)	−	No adverse event. Older age and higher global LV hemodynamic load were the main determinants of exercise capacity, which was not influenced by the conventional parameter of AS severity.
Levy et al., 2014 [38]	Prospective 28 ± 31 months	43	69 ± 13 years 72% men	CPET with upright bicycle. Ramp (20 W/min or 10 W/min) after a 1st min warm-up at 20 W	−	Exercise test was abnormal in 28% of patients. CPET better characterized revealed symptoms. Peak VO2 ≤ 14 mL/kg/min, VE/VCO2 slope > 34 were associated with abnormal exercise test.
Lumley et al., 2016 [23]	Cross-sectional	22 pts 38 control subjects	69 ± 8 years 82% men, 61 ± 10 years 74% men	Supine bicycle (25 + 25 W each 2nd min)	+	Exercise test during cardiac catheterization was safe and feasible. Ischemia in AS was not related to microvascular disease, but rather to abnormal cardiac-coronary coupling.
Masri et al., 2016 [20]	Retrospective 82.8 ± 39.6 months	533	66 ± 13 years 78% men	Treadmill (Bruce, modified Bruce, Cornell, Naughton)	+	No adverse event. Symptoms were revealed in 19% of patients.
Pérez del Villar et al., 2017 [22]	Cross-sectional	20	77 ± 16 years 85% women	Ergometer, 30° lateral decubitus (25 + 25 W each 3rd min)	+	Exercise testing was safe and feasible. Invasive hemodynamic monitoring showed that the aortic valve was highly dynamic and flow dependent.
Saeed et al., 2018 [16]	Retrospective 34.9 ± 35.1 months	316	65 ± 12 years 67% men	Treadmill (Bruce, modified by 2 warm-up stages)		No adverse event. Revealed symptoms in 29% of patients. lower peak SBP and rapid early rise in heart rate were associated with a higher risk of revealed Symptoms, while the use of antihypertensive treatment was associated with a lower risk of revealed symptoms. Serial testing had incremental prognostic value over baseline test.

CPET, cardiopulmonary exercise testing; EF, ejection fraction; SPAP, systolic pulmonary artery pressure.

## Data Availability

Not applicable.
